# Unveiling the Hidden Regulators: The Impact of lncRNAs on Zoonoses

**DOI:** 10.3390/ijms25063539

**Published:** 2024-03-21

**Authors:** Bojie Xu, Yujuan He, Ruicheng Yang, Junmin Li, Xiangru Wang

**Affiliations:** 1 School of Public Health, Health Science Center, Ningbo University, Ningbo 315211, China; xubojie@nbu.edu.cn; 2 State Key Laboratory for Managing Biotic and Chemical Threats to the Quality and Safety of Agro-Products, Institute of Plant Virology, Ningbo University, Ningbo 315211, China; heyujuan@nbu.edu.cn (Y.H.); lijunmin@nbu.edu.cn (J.L.); 3 National Key Laboratory of Agricultural Microbiology, College of Veterinary Medicine, Huazhong Agricultural University, Wuhan 430070, China; yangruicheng@mail.hzau.edu.cn

**Keywords:** zoonoses, long non-coding RNAs, pathogens, animals

## Abstract

Zoonoses are diseases and infections naturally transmitted between humans and vertebrate animals. They form the dominant group of diseases among emerging infectious diseases and represent critical threats to global health security. This dilemma is largely attributed to our insufficient knowledge of the pathogenesis regarding zoonotic spillover. Long non-coding RNAs (lncRNAs) are transcripts with limited coding capacity. Recent technological advancements have enabled the identification of numerous lncRNAs in humans, animals, and even pathogens. An increasing body of literature suggests that lncRNAs function as key regulators in zoonotic infection. They regulate immune-related epigenetic, transcriptional, and post-transcriptional events across a broad range of organisms. In this review, we discuss the recent research progress on the roles of lncRNAs in zoonoses. We address the classification and regulatory mechanisms of lncRNAs in the interaction between host and zoonotic pathogens. Additionally, we explore the surprising function of pathogen-derived lncRNAs in mediating the pathogenicity and life cycle of zoonotic bacteria, viruses, and parasites. Understanding how these lncRNAs influence the zoonotic pathogenesis will provide important therapeutic insights to the prevention and control of zoonoses.

## 1. Introduction

Zoonoses constitute a class of diseases naturally transmitted between humans and vertebrate animals [1]. They are typically classified based on their epidemiological characteristics into three categories: endemic zoonotic diseases, prevalent in numerous regions and impacting both human and animal health (e.g., brucellosis, rabies); epidemic zoonoses, which occur sporadically over time and space (e.g., H1N1 influenza); and emerging or re-emerging zoonotic diseases (e.g., Ebola hemorrhagic fever, Nipah virus encephalitis, severe acute respiratory syndrome) [2], the latter two of which pose a greater threat due to recent outbreaks. Given the inseparable connection among humans, animals, and their respective environments, it is inevitable for pathogens to spread between humans and animals. Approximately 60% of known infectious diseases and up to 75% of emerging infectious diseases are zoonotic in origin [3,4], making them a significant global public health concern with serious implications for human health and socio-economic well-being. The spillover of zoonotic pathogens is determined by a series of processes, and the probability of spillover is determined by the interactions among the barriers and the associated bottlenecks that might prevent cross-species transmission. These include: (1) pathogen dynamics in reservoir hosts, (2) pathogen release from reservoir hosts, (3) pathogen survival or dispersal outside of reservoir hosts, (4) exposure of the recipient host to the pathogen, (5) structural and physical barriers within the recipient host, (6) innate immune response and molecular compatibility, and (7) the replication and life cycle of the pathogen within the recipient host [5,6]. Failure to overcome any of these obstacles prevents pathogen spillover. Despite an extensive literature on emerging zoonotic diseases, there remains a gap of systematic understanding of the mechanisms underlying cross-species transmission of pathogens, especially the molecular mechanisms behind the pathogen’s ability to overcome the last three critical barriers. Therefore, comprehending how pathogens breach host barriers, evade the host immune system, and replicate during the process, along with identifying key regulatory molecules, is crucial for elucidating the cross-species transmission of zoonotic diseases. This knowledge is vital for developing preventive and control strategies for these zoonoses.

The advancement of high-throughput sequencing technologies has illuminated the extensive presence of non-coding RNAs (ncRNAs) in the transcriptomes of humans, animals and even pathogens. In humans, for instance, less than 3% of transcripts are responsible for encoding proteins [7], indicating that the majority of the transcriptional output of the human genome is non-coding. Among these ncRNA entities, a significant type is the long non-coding RNAs (lncRNA), which are characterized by transcripts exceeding 200 nucleotides in length and generally do not encode proteins [8]. Initially dismissed as “transcriptional noise”, subsequent research has established lncRNAs as crucial regulatory elements in numerous biological processes [9]. To date, over 90,000 long non-coding transcripts have been annotated in humans alone, with this figure undergoing continuous refinement [10,11]. These functional lncRNAs play key roles in uncovering the complexities of biological systems, and understanding the mechanisms of action of functional lncRNAs contributes to a deeper understanding of the hidden secrets within specific biological processes. According to the rules established by lncipedia, lncRNAs in mammals can be classified into five categories based on their genomic proximity to protein-coding genes: (1) sense overlapping lncRNA (also termed intragenic lncRNA), transcribed from the sense strand with complete or partial overlapping with coding genes; (2) antisense lncRNA, transcribed from the antisense strand of coding genes; (3) intronic lncRNA, transcribed entirely from introns of coding genes; (4) bidirectional lncRNA, transcribed from a promoter of a coding gene, yet in the opposite direction; and (5) intergenic lncRNA, transcribed from the intergenic regions between protein-coding genes [10,12,13]. The regulatory mechanisms of these lncRNAs are diverse, with most being closely associated with their subcellular localization [14,15]. Typically, in the nucleus, lncRNAs are involved in epigenetic and transcriptional regulation [16,17], including chromatin modifications, and transcriptional modulation by recruiting, binding or antagonizing transcription factors [18,19]. Conversely, in the cytoplasm, lncRNAs are primarily influenced by post-transcriptional events, including maintaining the stability of mRNA, sponging microRNAs to influence gene silencing, and regulating the integrity and activity of protein complexes [20,21]. Additionally, certain cytoplasmic lncRNAs can indirectly impact transcription by interacting with transcription factors [22]. Besides conventional regulatory mechanisms, recent studies have identified open reading frames (ORFs) within some cytoplasmic lncRNAs, suggesting they can encode functional peptides, and perform significant roles in various pathological processes [23,24,25]. Studies have indicated that lncRNAs are present not only in nonspecific barriers, such as endothelial cells and epithelial cells, but also in immune cells, such as macrophages, T cells, monocytes, neutrophils, dendritic cells, and B cells [26,27,28]. Some lncRNAs exhibit significant differential expression in response to pathogen infection [29,30], highlighting their potential association with infectious diseases, particularly zoonoses. The objective of this review is to provide a comprehensive overview of the roles played by lncRNAs in zoonotic diseases, a group of infectious diseases of considerable global importance, and to offer insights into emerging connections between lncRNAs and the transmission of zoonotic pathogens, thereby providing a new perspective for exploring preventive and therapeutic interventions against zoonotic diseases.

## 2. LncRNAs in Bacterial Zoonoses

Statistics reveal that bacterial infections represent the largest proportion of zoonotic diseases. Taking bovine-origin zoonotic pathogens as an example, bacterial agents account for 42%, compared to 22% viral, 29% parasitic, 7% fungal, and others [31]. Bacteria in this context encompasses both Gram-negative and Gram-positive bacteria, and their transmission routes include foodborne/fecal-oral transmission, occupational exposure, transmission through animal bites/scratches, transmission through contaminated environments, and vector-borne transmission [32]. The widespread adoption of antibiotics has been effective in controlling bacterial diseases for an extended period, minimizing the occurrence of public health crises. However, the increasing prevalence of antibiotic-resistant strains, especially multidrug-resistant organisms, in recent years [33] highlights the underestimated harm of bacterial zoonotic diseases. Therefore, research of the underlying transmission and pathogenesis mechanisms is urgently required. There is compelling evidence that lncRNAs exhibit significant differential expression in response to bacterial infections. Pathogens such as *Mycobacterium tuberculosis*, *Escherichia coli*, *Brucella*, *Salmonella enterica*, *Pseudomonas aeruginosa*, *Listeria monocytogenes*, and *Staphylococcus aureus* have been shown to induce changes in lncRNA expression, suggesting their vital regulatory roles in bacterial zoonoses (Table 1) [29]. These findings indicate the potential of lncRNAs as targets for understanding and managing zoonotic bacterial infections.

### 2.1. Tuberculosis

Tuberculosis, caused by the facultative intracellular bacterium *Mycobacterium tuberculosis* (Mtb), triggers a cellular immune response, and is predominantly mediated by CD4+ T cells, which in turn activate macrophage effector functions. Mtb is adept at persisting and multiplying within macrophages, leading to severe infections in hosts [34]. Moreover, CD8+ T cells are also recognized as critical defenders against Mtb, contributing to the host’s immune defense [35]. Studies have shed light on the significant roles of lncRNAs in regulating host responses to Mtb infection and facilitating the pathogen’s intracellular survival. Notably, lncRNAs such as *lncRNA-CD244*, nuclear paraspeckle assembly transcript *1 (NEAT1)*, *XLOC_012582*, *PCED1B-AS1*, *MIR3954HG*, *lincRNA-EPS*, *lincRNA-Cox2* and *lnc-EST12* are implicated in the processes of Mtb invasion and the initiation of immune responses [29,36,37,38,39,40]. Mechanically, for example, a *lncRNA-CD244* induced by CD244 during tuberculosis infection recruits enhancer of zeste homolog 2 (EZH2), an inducer of H3K27 methylation, to the infg/tnfa promoter, promoting H3K27 trimethylation, suppressing the expression of IFN-γ/TNF-α in CD8+ T cells, and exacerbating the infection [36]. Furthermore, lncRNAs, such as differentiation antagonizing non-protein coding RNA (*DANCR*), *MIR99AHG*, X inactive specific transcript (*XIST*), and myocardial infarction associated transcript (*MIAT*), have been identified as facilitators of Mtb intracellular survival, among which *MIR99AHG* is seen to promote Mtb intracellular persistence within macrophages by interacting with hnRNPA2/B1 and regulating host inflammatory response [41,42,43,44]. Other lncRNAs, such as *LINC00870*, colon cancer associated transcript 1 (*CCAT1*), and *LOC152742*, are emerging as potential novel biomarkers for the diagnosis of tuberculosis [45,46]. Their differential expression in response to Mtb highlights their potential utility in improving diagnostic accuracy and contributing to improved understanding of the disease’s pathogenesis.

### 2.2. Colibacillosis

*Escherichia coli* (*E. coli*), among the most prevalent bacteria in nature, poses an urgent threat to human and animal health, with infections affecting the intestines, urinary tract, blood, and brain. In intestinal infections, research has highlighted that Shiga toxin-producing *E. coli* infections result in the differential expression of 702 lncRNAs within human intestinal epithelial cells [47,48]. Specifically, the F18 *E. coli* strain, known for causing intestinal infections and diarrhea, is influenced by lncRNA *FUT3-AS1*. *FUT3-AS1* regulates the expression of FUT3 through H4K16ac modification or the miR-212/FUT3 pathway, and FUT3 in turn controls the invasion of *E. coli* into intestinal epithelial cells, ultimately leading to an enhancement of *E. coli* infection in the host. [49]. In extraintestinal infections, such as those affecting the brain, significant alterations in lncRNA expression patterns have been observed. Astrocytes and human brain microvascular endothelial cells show differential transcription of 74 and 289 lncRNAs, respectively, during *E. coli* infections [50,51]. Among these, *lncRSPH9-4* functions as a regulatory sponge, maintaining blood–brain barrier integrity by competitively interacting with miR-17-5p and matrix metallopeptidase 3 (*MMP3*) [52]; while *lncC11orf54-1* and *DDIT-AS1* mediate central nervous system inflammatory responses by interacting with interleukin 1 receptor associated kinase 1 (IRAK1) and DNA damage inducible transcript 4 (*DDIT4*) mRNA, respectively [51,53]. In mammary infections, *XIST* plays a protective role against damage from excessive inflammatory responses via the NF-κB/NLRP3 inflammasome pathway [54]. Additionally, lipopolysaccharide (LPS), the major virulence factor of *E. coli*, is also reported to alter lncRNA expression patterns, and detailed studies suggest that the best characterized lncRNAs, including HOX transcript antisense RNA (*HOTAIR*), SOX2 overlapping transcript (*SOX2OT*) and metastasis associated lung adenocarcinoma transcript 1 (*MALAT1*), are considered the essential regulator of LPS-related inflammation, as they all work as endogenous miRNA sponge and eventually affect the level of effector molecules [55,56,57]. This underscores the essential regulatory roles of lncRNAs in both intestinal and extraintestinal *E. coli* infections, as well as in the broader inflammatory response.

### 2.3. Brucellosis

*Brucella*, a prevalent zoonotic pathogen in veterinary medicine, also poses occasional but significant risks to humans. Similar to Mtb, *Brucella* is also a facultative intracellular bacterium capable of causing systemic infections in the host, leading to a range of symptoms, including undulant fever, endocarditis, arthritis, osteomyelitis, and reproductive disorders [58]. Evidence suggests that the pathogenesis of *Brucella* is attributed to the bacterial surviving intracellularly within both the phagocytic and non-phagocytic cells of its hosts [59]. Macrophages, which serve as the primary target cells for *Brucella*, are infected with the *Brucella* in studies involving RAW264.7 cells. Subsequent analyses reveal that 8, 6, 130, and 94 lncRNAs are differentially expressed at 4, 8, 24 and 48 h post-infection, respectively. Among them, *lnc_000428* promotes the intracellular replication of *Brucella* within macrophages, leading to stealthy and sustained spread [60]. Conversely, the infection leads to a decreased expression of another lncRNA, *Gm28309*, which activates inflammatory pathways and ultimately enhances bacterial clearance in macrophages. This regulatory process is initiated by activating NF-κB signaling through the modulation of the *Gm28309*/miR-3068-5p/κB-Ras2 axis [61]. Additionally, studies have identified potential biomarkers for *Brucella* infection, including *linc-MAF-4*, *IFNG-AS1*, and others [62,63].

### 2.4. Salmonellosis

*Salmonella* is recognized as one of the major food-borne zoonotic pathogens, with *Salmonella typhimurium* (*S. typhimurium*) being the most common cause of human infections. With increasing demands for food consumption, concentrated farming of livestock and poultry, and the rise of antibiotic-resistant strains, the incidence of *S. typhimurium* infections in humans is steadily increasing. *S. typhimurium* is an enteroinvasive pathogen causing gastrointestinal symptoms such as diarrhea and vomiting; in severe cases, it can escalate to systemic infections via the lymphatic and bloodstream, affecting multiple organs [64]. *NEAT1*, a lncRNA, emerges as a biomarker for *S. typhimurium* infection, and is significantly upregulated during infection, serving as a differential marker from other Salmonella strains or heat-inactivated *S. typhimurium* [65]. Another lncRNA, *LNCGM1082*, induced by *S. typhimurium* in macrophages serves as a molecular scaffold, mediating the binding of protein kinase Cδ with the inflammasome NLRC4 to induce the phosphorylation and activation of NLRC4, thereby promoting the host immune defense against infection [66]. The T cell-derived enhancer-like lncRNA termed *NeST* (also known as *IFNG-AS1*) can alter the host susceptibility to *S. typhimurium* by regulating the epigenetic marking of IFNγ-encoding chromatin, affecting the expression of related genes. Hosts deficient in *IFNG-AS1* exhibit increased vulnerability to fatal infection with *Salmonella enteritidis*, highlighting the crucial role of *IFNG-AS1* in the host defense against *Salmonella* infections [67]. In addition to its gastrointestinal impact, *S. typhimurium* can also affect the central nervous system, with studies, like those by Zou et al., indicating that lncRNA *TVX1* can mitigate *S. typhimurium*-induced microglial inflammation [68].

### 2.5. Pseudomonas aeruginosa Infection

*Pseudomonas aeruginosa* (*P. aeruginosa*), an opportunistic pathogen, is notorious for causing severe infections in immunocompromised individuals, particularly leading to hospital-acquired pneumonia and respiratory failure [69]. The escalating resistance of *P. aeruginosa* to diverse antibiotics serves to underscore its deleterious impact. Recent studies have illuminated the role of lncRNAs in the host response to *P. aeruginosa* infection, revealing intricate mechanisms by which these pathogens evade immune defenses and proliferate within the host. Infection with *P. aeruginosa* has been shown to suppress the expression of lncRNAs maternally expressed 9 (*MEG9*) and bladder cancer-associated transcript 1 (*BLACAT1*) in bronchial epithelial cells [70]. Additionally, maternally expressed 3 (*MEG3*), another lncRNA, is downregulated in *P. aeruginosa*-infected lungs through a TLR4/NF-κB-dependent pathway. *MEG3* normally acts by competitively binding to miR-138 alongside *IL-1β* mRNA. The suppression of *MEG3* leads to decreased levels of IL-1β, disturbing the immune balance during infection and affecting the proliferation of *P. aeruginosa* within the host [71]. Moreover, a small bacterial signaling molecule, termed N-3-(oxododecanoyl)-L-homoserine lactone (3-O-C12-HSL), which is secreted by *P. aeruginosa*, has been observed to enhance the expression of the lncRNA negative regulator of interferon response (*NRIR*). This, in turn, hinders the maturation of monocyte-derived dendritic cells and suppresses host immune responses [72].

### 2.6. Listeriosis

*Listeria monocytogenes* (*L. monocytogenes*) stands out as the only species of *Listeria* that is pathogenic to both humans and other vertebrates [73], and this environmental stress-tolerant pathogen is associated with serious public health and economic implications. It ranks among the most lethal foodborne pathogens, causing severe infections in immunocompromised individuals, such as septicemia, miscarriage, meningitis, and encephalitis [74,75]; in healthy individuals, meanwhile, it establishes latent infections, causing gastroenteritis [76]. Being a facultative intracellular pathogen, its pathogenicity is evident in its ability to survive and proliferate within host cells post-phagocytosis, as well as its immune-evasion capacity against cellular immune responses [77,78]. Specifically, in macrophages and dendritic cells, a lncRNA termed *lincRNA-EPS* binds to chromatin and interacts with heterogeneous nuclear ribonucleoprotein L (hnRNPL), a member of a large family of heterogeneous ribonucleoproteins, to alter nucleosome positioning and repress the transcription of immune-related genes (IRGs). The substantial reduction of *lincRNA-EPS* during *L. monocytogenes* infection leads to an enhanced inflammation, oxidative stress and lethality in hosts, highlighting its pivotal function in bolstering host defense responses against infections [79,80]. Similarly, *lincRNA-Cox2* is also characterized as regulating the inflammatory response and the macrophages’ function [81]. Furthermore, *L. monocytogenes* induces the production of lncRNA *AS-IL-1α*, which recruits RNAPII to the IL-1α promoter, thus resulting in heightened host inflammatory levels [82]. Additionally, *L. monocytogenes*-induced miR-1 targets non-coding RNA suppressor of Stat1 (*Sros1*) for degradation, relieving the inhibitory effect of *Sros1* on CAPRIN1/STAT1/IFN-γ axis and facilitating the bacterial clearance by the host [83]. Intriguingly, *L. monocytogenes* itself harbors lncRNAs, including a series of long antisense non-coding RNAs (lasRNAs), such as *las0333*, *las0936*, *las0996*, *las1136*, and *las2677*, which potentially affect the bacterium’s intracellular survival within eukaryotic hosts [84]. This highlights the complexity of lncRNA-mediated regulatory mechanisms in Listeriosis.

### 2.7. Staphylococcosis

In veterinary clinical settings, the *Staphylococcus* genus, characterized by its grape-like cluster appearance, comprises a diverse array of opportunistic pathogenic bacteria. Among these, the coagulase-positive *staphylococci* constitute the most pathogenic species *Staphylococcus aureus* (*S. aureus*). This historically emerging zoonotic pathogen poses substantial public health and veterinary challenges [85]. A critical concern with *S. aureus* is its antibiotic resistance—this is especially noted in methicillin-resistant *S. aureus* (MRSA), which is associated with a variety of severe infections ranging from food poisoning to more severe conditions, like endocarditis, pneumonia, otitis media, osteomyelitis, and skin or soft tissue infections [86]. Alpha-hemolysin, a critical virulence factor of *S. aureus*, induces hemolysis, cell lysis, and apoptosis, and its regulation is mediated by the two-component system [87,88]. Within this context, a prokaryotic lncRNA named *SSR42*, regulated by the global regulator Rsp, has been identified in *S. aureus*. The upregulation of *SSR42* positively regulates the two-component system SaeRS, thereby promoting alpha-hemolysin expression, enhancing *S. aureus* pathogenicity, and potentially influencing cross-species transmission [89,90]. From the host’s perspective, the host-derived bovine mastitis-related long non-coding RNA (*BMNCR*) triggers an inflammatory response in bovine mammary glands through the miR-145/*CBFB* axis, bolstering the autoprotective mechanism against *S. aureus* infection [91]. In addition, in bovine mammary epithelial cells, the antisense lncRNA *LRRC75A-AS* protects leucine rich repeat containing 75A (*LRRC75A*) mRNA from degradation by binding its coding sequence (CDS) region. During *S. aureus* infection, downregulation of *LRRC75A-AS1* acts as a protective mechanism, preserving tight junction proteins and impeding bacterial invasion [92].

**Table 1:** LncRNAs in the regulation of bacterial zoonoses.

Pathogen	LncRNA	Category	Function or Mechanism	Reference
Mtb	*lncRNA-CD244*	Host antisense lncRNA	Regulate T-cell responses against TB infection	[36]
	*NEAT1*	Host intergenic lncRNA	Regulate the inflammatory responses in macrophages	[37,93]
	*XLOC_012582*	Host intergenic lncRNA	Regulate the expression of SOCS3	[38]
	*PCED1B-AS1*	Host antisense lncRNA	Modulate macrophage apoptosis and autophagy by targeting miR-155	[39]
	*lincRNA-EPS*	Host intergenic lncRNA	Regulate apoptosis and autophagy of macrophages via JNK/MAPK signaling	[94]
	*lincRNA-Cox2*	Host intergenic lncRNA	Regulate macrophage apoptosis	[95]
	*lnc-EST12*	Host intergenic lncRNA	Regulate anti-Mtb innate immunity through FUBP3	[40]
	*DANCR*	Host intergenic lncRNA	Restrain intracellular survival of Mtb via miR-1301-3p and miR-5194	[41]
	*XIST*	Host intergenic lncRNA	Promote the polarization of macrophages to the M1 phenotype via miR-125b-5p/A20/NF-κB axis	[42]
	*MIAT*	Host intergenic lncRNA	Regulate autophagy and antimicrobial responses	[43]
	*MIR99AHG*	Host intergenic lncRNA	Promote Mtb growth by regulating inflammation and macrophage polarization	[44]
	*LINC00870*	Host intergenic lncRNA	Biomarker	[45]
	*CCAT1*	Host intergenic lncRNA	Biomarker	[45]
	*LOC152742*	Host intergenic lncRNA	Biomarker	[45]
	*MIR3945HG*	Host intergenic lncRNA	Biomarker	[46]
*E. coli*	*FUT3-AS1*	Host antisense lncRNA	Modulates *E. coli* susceptibility via histone H4 modifications	[49]
	*lncRSPH9-4*	Host sense overlapping lncRNA	Disrupt endothelial barrier via miR-17-5p/*MMP3* axis	[52]
	*lncC11orf54-1*	Host intronic lncRNA	Modulate neuroinflammation responses	[51]
	*DDIT-AS1*	Host antisense lncRNA	Modulate DDIT4 expression and promote neuroinflammation responses	[53]
	*XIST*	Host intergenic lncRNA	Regulate NF-κB/NLRP3 inflammasome pathway	[54]
	*HOTAIR*	Host antisense lncRNA	Promote kidney injury in sepsis	[55]
	*SOX2OT*	Host sense overlapping lncRNA	Mitigate LPS-induced injuries in cardiomyocytes	[56]
	*MALAT1*	Host intergenic lncRNA	Regulate macrophage polarization	[57]
*Brucella*	*lnc_000428*	Host antisense lncRNA	Regulate Brucella intracellular replication	[60]
	*Gm28309*	Host intronic lncRNA	Regulate inflammatory and anti-Brucella responses via NF-κB/NLRP3 signaling	[61]
	*linc-MAF-4*	Host intergenic lncRNA	Biomarker	[62]
	*IFNG-AS1*	Host intergenic lncRNA	Biomarker	[63]
*S. typhimurium*	*LNCGM1082*	Host intergenic lncRNA	Activate NLRC4 and induce resistance to *S. typhimurium*	[66]
	*NeST* *(IFNG-AS1)*	Host intergenic lncRNA	Modulate host susceptibility to pathogens by altering epigenetic marking of IFNγ-encoding chromatin	[67]
	*TVX1*	Host intergenic lncRNA	Attenuated *S. typhimurium*-induced microglial inflammation	[68]
	*NEAT1*	Host intergenic lncRNA	Biomarker	[65]
*P. aeruginosa*	*MEG3*	Host intergenic lncRNA	Influence the proliferation of *P. aeruginosa* by miR-138/*IL-1β* axis	[71]
	*NRIR*	Host intergenic lncRNA	Affect the maturation of dendritic cell and the activation of T cell	[72]
	*MEG9*	Host intergenic lncRNA	Biomarker	[70]
	*BLACAT1*	Host intronic lncRNA	Biomarker	[70]
*L. monocytogenes*	*lincRNA-EPS*	Host intergenic lncRNA	Impair the host defense against *L. monocytogenes* infection	[79,80]
	*lincRNA-Cox2*	Host intergenic lncRNA	Regulate migration and phagocytosis of macrophages	[81]
	*AS-IL-1α*	Host antisense lncRNA	A regulator of innate immune response by regulating *IL-1α* transcription	[82]
	*SROS1*	Host intergenic lncRNA	Promote IFN-γ-STAT1-mediated innate immunity	[83]
	*lasRNAs*	Pathogen-derived lncRNA	Represent a regulatory pattern that connect adjacent genes with opposing functions	[84]
*S. aureus*	*BMNCR*	Host intronic lncRNA	Influence the proliferation and apoptosis of epithelial cells	[91]
	*LRRC75A-AS*	Host antisense lncRNA	Regulate the expression of tight junctions and affect inflammation	[92]
	*SSR42*	Pathogen-derived lncRNA	Modulate the expression of several virulence factors	[89,90]

## 3. LncRNAs in Viral Zoonoses

Although viruses do not constitute the majority of zoonotic diseases, they are often responsible for explosive outbreaks in humans due to their high variability and the scarcity of specific treatments. This leads to a series of public health events, adversely affecting health, socio-economic, and political landscapes. The last four pandemics were all attributed to viruses, making them a globally prioritized zoonotic disease. In the realm of molecular biology, lncRNAs have emerged as crucial players in the spillover and pathogenic mechanisms of zoonotic viruses. Extensive research has documented the involvement of lncRNAs across a variety of zoonotic viral infections, spanning several families such as *Rhabdoviridae* (e.g., rabies virus), *Filoviridae* (e.g., Ebola virus), *Flaviviridae* (e.g., Japanese encephalitis and Dengue viruses), *Poxviridae* (e.g., Monkeypox virus), *Retroviridae* (e.g., HIV), and various influenza viruses, coronaviruses and herpesviruses (Table 2) [96,97].

### 3.1. Rabies

Rabies, an ancient and deadly zoonotic disease caused by the Rabies virus (RABV), results in over 59,000 deaths annually worldwide [98]. Despite being vaccinepreventable, rabies progresses rapidly and is almost invariably fatal once clinical symptoms manifest [99]. RABV is a non-segmented negative-stranded RNA virus belonging to the *Lyssavirus* genus of the *Rhabdoviridae* family in the order *Mononegavirales*. Most RABV infections initiate from a dermal or muscular wound, which means RABV replicates locally in muscle tissue and then enters peripheral neurons at axon termini, requiring long distance axonal transport and trans-synaptic spread between neurons for the infection of the central nervous system [100,101]. Studies have identified a RABV-inducible lncRNA in neuronal cells, known as *EDAL*, which is short for EZH2 Degradation-Associated lncRNA. *EDAL* interacts with the T309 region of the *EZH2* gene, diminishing *EZH2* levels and its enzymatic output H3K27me3 via the lysosomal pathway. This ultimately hinders the replication of the RABV by regulating the transcription of corresponding peptides, highlighting the pivotal role of *EDAL* as a prominent restriction factor in the cross-species spillover of RABV [102]. Moreover, the introduction of *EDAL* expression in engineered RABV substantially reduces its pathogenicity following nasal infection [103].

### 3.2. Ebola Virus Disease

Ebola virus disease (EVD), triggered by EBOV, is an acute and often fatal illness. An epidemic occurring in West Africa stemmed from a single zoonotic transmission event to a two-year-old boy in Meliandou, Guinea, and led to subsequent human-to-human transmission [104]. EVD is characterized by hemorrhagic fever, gastrointestinal symptoms, and multiple organ dysfunction syndrome with high fatality rates [105]. Despite the extensive research of EVD, the role of lncRNAs in its pathogenesis had remained unexplored until a recent single-cell sequencing study shed light on this area. This study identified 3979 unannotated novel lncRNAs in EBOV-infected rhesus monkeys, with a significant number showing differential expression in response to EBOV infection, including the upregulation of lncRNAs small nucleolar RNA host gene 6 (*SNHG6*) and *LINC00861*, and the downregulation of lncRNA *NEAT1* [106]. This investigation further elucidates the mechanisms underlying the tissue-specific characteristics of lncRNAs, based on single-cell analyses. Fundamentally, lncRNAs are present exclusively within certain specific cell types, which explains their apparent predilection for particular tissues. This indicates that the tissue specificity of lncRNAs is not due to their low-level expression across all cell types within certain tissues but rather because they are expressed in a limited number of cell types. Detailed mechanisms showed that lncRNAs harbor fewer transcription factor binding sites and higher chromatin repressive marks in their promoter regions, thereby decreasing the probability of transcription rather than the strength of transcription. This study not only identifies potential lncRNA markers in the context of EBOV infection that underlie the involvement of lncRNAs in immune regulations but also addresses the question of how lncRNAs differentially respond to viral infection at single-cell resolution.

### 3.3. Flavivirus Infection

Both Dengue fever and Japanese encephalitis are mosquito-borne acute encephalitis syndromes caused by DENV and JEV, respectively. These viruses, belonging to the *Flaviviridae* family, are single-stranded positive-sense RNA-enveloped viruses with zoonotic characteristics [107,108]. DENV transmission occurs through mosquito bites, proliferating throughout the body with white blood cells and triggering signaling protein production, resulting in the manifestation of symptoms such as pain and fever. This process also increases vascular permeability, causing hemorrhage and multiorgan involvement [109]. JEV, on the other hand, followed by mosquito bites, enters the mononuclear-phagocyte system and undergoes replication [110]. It subsequently leads to a robust viremia in individuals due to weakened immune systems, allows the penetration of the blood–brain barrier, and causes extensive meningoencephalitis [111,112]. Research has linked the lncRNA *NEAT1* with DENV proliferation. Knocking down *NEAT1* enhances the expression of interferon alpha-inducible protein 27 (IFI27) through the RIG-I pathway, thereby inhibiting the DENV replication [113]. Another DENV-induced lncRNA, ERG-Associated lncRNA (*ERGAL*) competitively binds to miR-183-5p, mitigating the inhibitory effect of miR-183-5p on VE-cadherin, and claudin-5, which are important markers of blood–brain barrier (BBB) function, thereby enhancing the integrity of the BBB. In addition, *ERGAL* reduces early apoptosis of endothelial cells and facilitates cytoskeleton remodeling, thereby improving the blood–brain barrier stability and restricting DENV brain invasion [114]. As for JEV infection, an increased expression of *lncRNA-SUSAJ1* is observed as being regulated by the neuroinflammatory inducer CCR1/SP. *LncRNA-SUSAJ1*, in turn hampers JEV replication and interrupts the transmission of JEV [115,116]. In addition, several broad-spectrum regulatory lncRNAs, such as *JINR1*, *ZAP-IT1*, *MALAT1*, and *Gm20559*, are characterized in flaviviral diseases [117,118,119,120]. For instance, the flavivirus-induced lncRNA *JINR1*, mediated by NF-κB, is considered to be the facilitator of EV/DENV/WNV replication. It functions by interacting with RNA-binding protein RBM10, and manipulating the expression of NF-κB target genes, such as glucose-regulated protein 78 (*GRP78*) [117]. Another lncRNA *ZAP-IT1* is however induced by type I IFN, and is recognized as a negative regulator for flavivirus infection, and as inhibiting the replication of ZIKV, DENV, JEV and vesicular stomatitis virus (VSV) in a type I IFN signaling independent manner [118]. Interestingly, from the perspective of pathogens, all members of the Flaviviridae are likely to produce lncRNAs in their infected cells, these lncRNAs are generated by the stalling and degrading of host exonuclease Xrn1 on viral RNA structures, which impacts viral replication, cytopathology as well as pathogenesis, opening up the door to new therapeutic targets for the development of broad-spectrum antiflaviviral therapeutics [121].

### 3.4. AIDS

Acquired Immunodeficiency Syndrome (AIDS), caused by HIV, emerged as a global pandemic since being initially reported by the U.S. CDC in the early 1980s. To date, it has infected over 80 million individuals worldwide, resulting in approximately 40 million deaths [122,123]. It is currently widely believed that HIV may be of multiple origins instead of a single one, having evolved from various simian immunodeficiency viruses (SIVs), with HIV-1 being the predominant type responsible for human transmission. Traceability analyses propose the hypothesis that HIV-1 originated from recurrent SIV spillover events that can be traced back to the early 20th century [124,125]. In the context of HIV infection, lncRNAs play pivotal roles in regulating immune responses. In HIV-infected individuals, lncRNA RUNX1 overlapping RNA (*RUNXOR*) promotes the proliferation of myeloid-derived suppressor cells (MDSCs) and regulates the expression of various immune inhibitory signaling molecules by targeting the transcription factor runt-related transcription factor-1 (RUNX1), leading to an immune suppression [126]. Similarly, HOXA transcript antisense RNA myeloid-specific 1 (*HOTAIRM1*) exhibits comparable functionality in inhibitory immune regulation [127]. Another lncRNA, termed growth arrest specific 5 (*GAS5*), is shown to control HIV replication through interaction with miR-873 [128]. Additionally, *GAS5* also controls miR-21 expression and regulates signaling molecules involved in DNA damage and cellular responses following T cell receptor stimulation, reversing T cell dysfunction and improving CD4+ T cell exhaustion incurred during HIV infection [129]. NF-kappaB interacting lncRNA (*NKILA*) inhibits HIV-1 replication and reactivation by suppressing HIV-1-long-terminal-repeat-driven transcription initiation in an NF-κB-dependent manner, which holds potential significance for elucidating the mechanisms underlying HIV transmission and latent infection [130].

### 3.5. Influenza

Influenza is a contagious respiratory disease caused by influenza viruses, which are classified into four genera: influenza A viruses (IAV), influenza B viruses (IBV), influenza C viruses (ICV), and influenza D viruses (IDV). These viruses, along with other arthropods or fish-associated genera, such as Thogotovirus, Quaranja virus, Sardinevirus, Mykissvirus, and Isavirus, collectively form the family *Orthomyxoviridae*. [131]. Only three, IAV, IBV and ICV, have so far been described in humans, while only Influenza A is commonly transmitted from animals to human and vice-versa [132,133,134]. Zoonotic influenza viruses occasionally infect humans, leading to various outcomes, ranging from mild conjunctivitis to severe pneumonia and even death [135]. Over the past few decades, there have been several spillover events involving influenza viruses, such as outbreaks of H5N1, H9N2, H1N1, H3N2, H7N9, and H9N2 [136]. In the context of lncRNA research, a database termed VirhostlncR has been developed by Rajesh Raju et al. [137], which compiles differential expression profiles of lncRNAs in viral infections, incorporating data on six lncRNAs relevant to influenza. Analysis of this database indicates that lncRNAs, like *LINC01191*, *DANCR*, breast cancer anti-estrogen resistance 4 (*BCAR4*), and *PSMB8-AS1*, are pivotal in modulating influenza replication and pathogenesis [138,139,140]. For instance, influenza viruses, such as H1N1, H5N1, H7N9, induce the expression of *LncRNA#61*, which disrupts viral invasion, RNA synthesis, and release through its four long arms, effectively curbing viral replication and enhancing host immune defense [141]. Subsequent investigations have provided additional evidence supporting the broad-spectrum antiviral properties of *LncRNA#61*, as well as the similar functionality observed in *LncRNA#45* [142]. Other lncRNAs, like cholesterol induced regulator of metabolism RNA (*CHROMR*), *lncNSPL*, and RIG-I-dependent antiviral response regulator RNA (*RDUR*), contribute to the host anti-influenza virus response in an IFN-dependent manner [143,144,145].

### 3.6. Herpesvirus Infection

Herpesviruses, belonging to the family *Orthoherpesviridae*, are double-stranded DNA viruses comprising 17 genera [146]. Among them, certain ones, including pseudorabies virus (PRV), monkey B virus, and Epstein–Barr virus (EBV) are recognized as zoonotic. Additionally, some other herpesviruses, such as avian Marek’s disease virus (MDV), human herpes simplex virus type-1 (HSV-1), and equine herpesvirus type 1 (EHV), are considered to possess cross-species transmission potential between humans and animals in specific cases, although the available evidence regarding the zoonotic capabilities of these viruses remains insufficient [147,148]. During PRV infection, the host-derived lncRNA, *lnc_000641*, is identified to modulate viral replication by inhibiting the JAK-STAT1 signaling pathway, thereby influencing the expression of type I IFN [149]. Similarly, lncRNA *lncA02830* also influences PRV multiplication through akin mechanisms [150]. In the context of EBV, associated primarily with laryngeal cancer, the high expression of lncRNA *H19* in EBV-positive individuals is significantly correlated with the occurrence of laryngeal cancer, likely via the transcriptional repressor EZH2 regulation [151]. Remarkably, herpes viruses themselves harbor lncRNAs within their genomes. In PRV, lncRNAs *NOIR1* and *NOIR2* are located in the inverted repeat (IR) region, while lncRNAs *PTO* and *PTO-US1* are located in the vicinity of the viral replication origin sequence oriS. Additionally, lncRNAs *CTO-S* and *CTO-L* are positioned in the vicinity of the oriL sequence, and lncRNA *AZURE* is situated at the boundary of the US-IR region [152]. Subsequently, studies have established a transcriptional interference network with the involvement of these viral lncRNAs and their neighboring genes, thereby exerting an intriguing epigenetic regulatory function. This suggests the pivotal involvement of viral-derived lncRNAs in the regulation of herpesvirus pathogenesis and potentially their spillover mechanisms.

### 3.7. Coronavirus Disease

Since the outbreaks of SARS and COVID-19, coronavirus diseases have garnered significant global attention. Belonging to the *Orthocoronavirinae* subfamily, coronaviruses encompass four genera: α coronavirus, β coronavirus, γ coronavirus, and δ coronavirus. These single-stranded positive-sense RNA viruses were first identified in cases of infectious bronchitis in chickens [153,154]. Throughout millennia of evolution, coronaviruses have continually crossed species boundaries, causing profound infections in a diverse range of species, including humans, mammals, and birds [155]. The spike (S) protein of coronaviruses is widely acknowledged as the primary determinant of tissue tropism and the cross-species transmission capacities [156]. To date, human-infective coronaviruses have been identified with α-CoV, such as HCoV-NL63, HCoV-229E, CCoV-HuPn-2018 of, as well as β-CoV such as HCoV-OC43, HCoV-HKU1, SARS-CoV, MERS-CoV, and SARS-CoV-2. Among these, SARS-CoV, MERS-CoV, and SARS-CoV-2 exhibit the highest pathogenicity, and are capable of causing severe respiratory distress syndrome and extrapulmonary manifestations [157,158]. Research indicates that the angiotensin converting enzyme 2 (ACE2) receptor for the S protein is closely associated with coronavirus invasion, with lncRNA *GAS5* regulating the expression of ACE2 through the *GAS5*/miRNA-200/*ACE2* axis, thereby affecting the invasion of SARS-CoV-2 into the host [159]. Another lncRNA, SNHG15, modulates the hostinvasion of SARS-CoV-2 in an ACE2 independent manner through interacting with RABL2A, an essential regulator of vesicular trafficking in human cells [160]. During SARS-CoV-2 infection, changes in lncRNA expression, such as the downregulation of PU.1-induced regulator of alarmin transcription (*PIRAT*) and upregulation of lung cancer associated transcript 1 (*LUCAT1*), have been found to alter the production of immune mediators, impacting the host systemic antiviral responses [161]. In CD8+ T cells, the SARS-CoV-2-induced lncRNA small nucleolar RNA host gene 15 (*SNHG15*) interacts with the vesicle transport protein Vps13D and regulates the IL-7 signaling pathway, promoting the generation of memory CD8+ T cells. Furthermore, a series of lncRNAs, such as *MALAT1*, *MEG3*, *XIST*, *ZFY-AS1*, and *TTTY14*, serve as noteworthy biomarkers for SARS-CoV-2 infection [162,163]. These lncRNAs exhibit some pathological impacts and present novel targets for the advancement of diagnostic and therapeutic approaches.

**Table 2:** LncRNAs in the regulation of viral zoonoses.

Pathogen	LncRNA	Category	Function or Mechanism	Reference
RABV	*EDAL*	Host intergenic lncRNA	Inhibit the replication of neurotropic virus	[102,103]
DENV (*Flaviviridae*)	*NEAT1*	Host intergenic lncRNA	Affect antiviral response and viral replication in dengue infection	[113]
	*ERGAL*	Host intergenic lncRNA	Promote stability and integrity of vascular endothelial barrier during DENV infection	[114]
JEV (*Flaviviridae*)	*SUSAJ1*	Host sense overlapping lncRNA	Inhibit JEV proliferation and replication	[115,116]
*Flaviviridae*	*JINR1*	Host intergenic lncRNA	Regulate viral replication and cell death	[117]
	*ZAP-IT1*	Host intronic lncRNA	Exert antiviral effect in an IFN-independent manner	[118]
	*MALAT1*	Host intergenic lncRNA	Potential antiviral function	[119]
	*Gm20559*	Host intergenic lncRNA	Modulate the expression of various pro-inflammatory cytokines during flavivirus infection	[120]
	*sfRNAs/ xrRNAs*	Pathogen-derived lncRNA	Impact viral replication	[121]
HIV	*RUNXOR*	Host sense overlapping lncRNA	Regulate multiple immunosuppressive signaling molecules	[126,164]
	*HOTAIRM1*	Host intergenic lncRNA	Increase levels of immunosuppressive molecules	[127]
	*GAS5*	Host antisense lncRNA	Control HIV replication, regulate the activity and longevity of CD4 T cells	[128,129]
	*NKILA*	Host antisense lncRNA	Inhibit HIV-1 replication by suppressing HIV-1 LTR promoter activity	[130]
Influenza viruses	*PSMB8-AS1*	Host antisense lncRNA	Promotes influenza virus replication	[137,138]
	*LINC01191* *(VIN)*	Host intergenic lncRNA	Regulate viral protein synthesis	[137,140]
	*DANCR*	Host intergenic lncRNA	Involved in respiratory infections and regulate inflammation	[137,139]
	*BCAR4*	Host intergenic lncRNA	Biomarker	[137]
	*LncRNA#61*	Host sense overlapping lncRNA	Suppress viral replication, mediate host immune responses	[141]
	*LncRNA#45*	Host intronic lncRNA	Function as a broad-spectrum antiviral factor	[142]
	*CHROMR*	Host antisense lncRNA	Restrict influenza virus replication by sequestering IRF2/IRF2BP2 complex	[143]
	*lncNSPL*	Host intergenic lncRNA	Influence influenza immune escape by modulating IFN-I expression	[144]
	*RDUR*	Host intergenic lncRNA	Regulate innate immunity against virus by controlling IFN-β and ISGs	[145]
PRV (*Orthoherpesviridae*)	*lnc_000641*	Host intergenic lncRNA	Influence PRV replication through JAK-STAT1 pathway	[149]
	*lncA02830*	Host intronic lncRNA	Affect PRV replication in a IFN-dependent manner	[150]
	*NOIR1/NOIR2*	Pathogen-derived lncRNA	Locate in the IR region of the PRV	[152]
	*PTO/PTO-US1*	Pathogen-derived lncRNA	Overlap with the oriS region of the PRV	[152]
	*CTO-S/CTO-L*	Pathogen-derived lncRNA	Function as TATA boxes in herpesviruses	[152]
	*AZURE*	Pathogen-derived lncRNA	Locate in the IR-US overlapping region of the PRV	[152]
EBV (*Orthoherpesviridae*)	*H19*	Host intergenic lncRNA	Biomarker	[151]
SARS-CoV-2	*GAS5*	Host antisense lncRNA	Affect SARS-CoV-2 invasion via GAS5/miRNA-200/*ACE2* axis	[159]
	*SNHG15*	Host intergenic lncRNA	Aid SARS-CoV-2 entry through RABL2A, facilitate memory CD8+ T cell production	[160]
	*PIRAT*	Host intergenic lncRNA	Modulate systemic antiviral responses to SARS-CoV-2	[161]
	*LUCAT1*	Host intergenic lncRNA	Modulate systemic antiviral responses to SARS-CoV-2	[161]
	*XIST*	Host intergenic lncRNA	Biomarker	[162]
	*ZFY-AS1*	Host antisense lncRNA	Biomarker	[162]
	*TTTY14*	Host intergenic lncRNA	Biomarker	[162]
	*MALAT1*	Host intergenic lncRNA	Biomarker	[163]
	*MEG3*	Host intergenic lncRNA	Biomarker	[163]

## 4. LncRNAs in Parasitic Zoonoses

Zoonotic parasites are significant pathogens found in both animals and humans, and continue to cause substantial morbidity and mortality worldwide, indicating that the efforts of drug administration and parasite eradication campaigns have not yet effectively addressed all parasites that are of significance to public health and veterinary medicine [165,166]. Recent studies have demonstrated that lncRNAs are of major importance in both parasites and hosts, exerting diverse functions throughout the parasitic infection process [167,168]. Herewith, we compiled and summarized the current knowledge concerning the role of lncRNA in parasitic diseases, such as malaria, Echinococcosis, schistosomiasis, cryptosporidiosis, and toxoplasmosis (Table 3).

### 4.1. Malaria

Malaria is a mosquito-borne infectious disease caused by a protozoan parasite that belongs to the genus *Plasmodium* [169]. It is interesting to note that, as a eukaryote, lncRNAs are widespread in *Plasmodium* itself. For example, more than 2500 lncRNA transcripts have been found in *Plasmodium falciparum* (*P. falciparum*) [170]. Among these, lncRNAs, a Pfgdv1 gene-derived antisense lncRNA named *gdv1*, regulates the expression of Pfgdv1, which functions as an inhibitor of sexual differentiation and ultimately modulates the sexual development of *Plasmodium* [171,172]. Two lncRNAs transcribed from the telomere-associated repetitive elements (*TARE*), *TARE-3-lncRNA* and *TARE-6-lncRNA*, are able to influence the intra-erythrocytic developmental cycle of Plasmodium. Due to the enrichment of binding sites for various transcription factors, it is therefore postulated that *TARE-lncRNAs* function by regulating neighboring genes [172,173,174]. Another class of var gen-specific lncRNAs can be incorporated into chromatin, and play a key role in the activation of var gene, which is able to encode variable antigens and enhance the virulence of *P. falciparum*. In addition, interfering with these var-specific lncRNAs leads to the down-regulation of the var gene and alters its epigenetic imprint, which results in a switching of expression to different var genes [175]. Recently, Gayani Batugedara et al., identified 1768 intergenic lncRNAs in *P. falciparum,* using deep sequencing and nascent RNA expression. They also demonstrated that a nuclear lncRNA, *lncRNA-ch14*, plays an important role in gametocyte development and in the infectivity of these gametocytes for mosquitoes, by recruiting histone demethylase and histone acetyl transferase to change the epigenetic state of the chromatin and activate the expression of these genes during sexual differentiation [170]. Thus, it will be important to elucidate the function *Plasmodium*-derived lncRNA to understand the pathogenicity and pathogenesis of *Plasmodium*. On the other hand, host-derived lncRNA plays a significant role in regulating the interaction between parasites and their host. Previous studies have reported that 291 lncRNAs were differentially expressed in *Plasmodium*-infected mice; *ENMSUSG00000111521.1*, *XLOC_038009*, *XLOC_058629*, and *XLOC_065676* are considered to be involved in malaria infection. These four lncRNAs function as regulators of host immunity by activating TGF-β/Smad2/3 signaling pathway [176]. Moreover, an abundant, ubiquitously expressed lncRNA *MALAT1* is found to modulate Maf/IL-10 axis in CD4+ T cells by functioning as a negative regulator of cellular immune response upon *Plasmodium* infection. Knockout of the *MALAT* leads to an activated macrophage and reduced parasite loads [177].

### 4.2. Schistosomiasis

Schistosomiasis is one of the most serious zoonoses caused by *schistosomes*, which frequently causes intestinal symptoms, urogenital symptoms, and other systemic symptoms such as fever [178]. Similar to Malaria, lncRNAs are present both in the pathogen level and the host level during Schistosomiasis. Silveira et al., have identified 16583 lncRNAs in *Schistosoma mansoni* (*S. mansoni*). Among these, *SmLINC101519*, *SmLINC175062*, and *SmLINC110998* are correlated with the motility of adult worms, which further influences worm burden and egg hatching [179]. Another study reported 5-azacytidine, a potential antiparasitic agent, was considered to affect lncRNA levels in *S. mansoni* and be involved in *S. mansoni* reproductive biology [180]. Moreover, a total of 3033 potential lncRNAs are identified in *Schistosoma japonicum* (*S. japonicum*) [181]. Overall, these studies suggest lncRNAs may play an essential role in *Schistosoma* itself. On the host side, 759 and 789 differentially expressed lncRNAs are observed in liver and spleen of *S. japonicum* parasitized mice, respectively [182]. LncRNA Gm16685 is upregulated during *S. japonicum* infection, and participates in the pathogenesis throughout schistosomiasis by regulating miR-205-5p [183]. The involvement of lncRNA *H19* in *S. japonica* has also been corroborated, as it governs the hepatic reaction to Praziquantel therapy against *S. japonicum* infection through the *H19*/miR-130b-3p/*Cyp4a14* axis [184].

### 4.3. Cryptosporidiosis

*Cryptosporidium* is the causative agent of cryptosporidiosis, zoonotic cryptosporidiosis in humans, which usually causes diarrhea in immunocompromised individuals, and especially children [185]. The intestinal epithelial cells provide the first line of defense against *Cryptosporidium* infection. A study finds that a lncRNA, *U90926*, is induced by *Cryptosporidium parvum* (*C. parvum*) within the intestinal epithelial cells.This lncRNA targets the transcription of host defense genes and suppresses the epithelial antiparasitic response. Intriguingly, the *U90926* appears to be triggered by an RNA virus present in *Cryptosporidium* [186]. The pathogen-associated molecular patterns (PAMPs) related to signaling pathways, like NF-κB and IFN signaling pathways, are also implicated in lncRNA-dependent cryptosporidiosis pathogenesis. For instance, the NF-κB signaling-dependent lncRNAs *Nostrill*, *NR_045064* and *XR_001779380* regulate intestinal epithelial anti-*Cryptosporidium* defense through modulating downstream molecules NF-κB p65, NOS2/CSF2, or IFN-γ [187,188,189]; an up-regulated lncRNA *NR_033736*, in response to cryptosporidial infection, provides negative feedback regulation of type I IFN signaling through suppressing the transcript of type I IFN-controlled genes, thus influencing the epithelial innate defense against *C. parvum* [190].

### 4.4. Toxoplasmosis

Toxoplasmosis is a zoonotic parasitic disease caused by *Toxoplasma gondii* (*T. gondii*), an obligate intracellular apcomplexan parasite that is common in dogs and cats. Toxoplasmic encephalitis and ocular toxoplasmosis are two important manifestations of toxoplasmosis [191]. Studies report that 1522 lncRNAs are differentially regulated during infection with the high-virulence Type I *T. gondii* strain, versus 528 with the lessvirulent Type II *T. gondii* strain in mice; among these, host lncRNAs *Csf1-lnc* and *Socs2-lnc* are manipulated by *toxoplasma* rhoptry kinase ROP16, suggesting the strong influence of *Toxoplasma* on lncRNA expression patterns [192]. *T. gondii* is neurotropic and affects the function of nerve cells in the mouse brain, and researchers have found that differentially expressed *lncRNA147410.3* and *lncRNA-11496* elicited by *T. gondii* infection are involved in the processes of microglial apoptosis; among which *lncRNA147410.3* induces microglial apoptosis by positively regulating its target gene *Hoxb3*, while *lncRNA-11496* influences the biological processes of microglia by regulating the expression of the MEF2C/HDAC2 axis [193,194]. Moreover, in human foreskin fibroblast (HFF) cells, a total of 996 lncRNAs are identified as the differential expression candidates in response to *T. gondii* infection, of which one lncRNA, named *NONHSAT022487*, is able to stimulate the secretion of cytokines by suppressing the expression of UNC93B1, representing a novel mechanism by which *Toxoplasma* regulates lncRNA-mediated host immune signaling [195].

### 4.5. Echinococcosis

Echinococcosis is a serious zoonotic disease caused by the infections of *Echinococcus multilocularis* (*Em*) and *Echinococcus granulosus* (*Eg*) larvae, causing alveolar echinococcosis (AE) and cystic echinococcosis (CE), respectively [196]. In *Em*-infection models, 218 lncRNAs were differentially regulated in mice hepatocytes [197]. In *Eg*-infection models, a total of 649 differentially expressed lncRNAs were identified in splenic monocytic myeloid-derived suppressor cells, and 234 differentially expressed lncRNAs were found in human serum exosomes, which typically contain abundant DNA, mRNA, non-coding RNA, and proteins [198,199]. Functionally, *lncRNA028466* was considered the regulator of recombinant *Eg* antigen P29 (rEg.P29) vaccination-mediated Th1 protective immunity, with reduced *lncRNA028466* caused by rEg.P29 leading to the increased Th1 immune response and the lower IL-4 and IL-10 expression, suggesting the participation of lncRNAs in host–parasite interaction and CD4+ T cell differentiation [200].

**Table 3:** LncRNAs in the regulation of parasitic zoonoses.

Pathogen	LncRNA	Category	Function or Mechanism	Reference
*Plasmodium*	*GDV1*	Pathogen-derived lncRNA	Regulate sexual development	[171,172]
	*TARE-3-lncRNA/TARE-6-lncRNA*	Pathogen-derived lncRNA	Affect the intra-erythrocytic developmental cycle of *Plasmodium*	[172,173,174]
	*Var-specific lncRNA*	Pathogen-derived lncRNA	Enhance the virulence of *P. falciparum* by modulating var	[175]
	*LncRNA-ch14*	Pathogen-derived lncRNA	Regulate gametocyte development	[170]
	*ENMSUSG00000111521.1*	Host antisense lncRNA	Regulate host immunity by TGF-β/Smad2/3 signaling	[176]
	*XLOC_038009*	Host intergenic lncRNA	Regulate host immunity by TGF-β/Smad2/3 signaling	[176]
	*XLOC_058629*	Host intergenic lncRNA	Regulate host immunity by TGF-β/Smad2/3 signaling	[176]
	*XLOC_065676*	Host intergenic lncRNA	Regulate host immunity by TGF-β/Smad2/3 signaling	[176]
	*MALAT1*	Host intergenic lncRNA	Function as a negative regulator of cellular immune response	[177]
*Schistosoma*	*SmLINC101519*	Pathogen-derived lncRNA	Regulate the motility of adult worms	[179]
	*SmLINC175062*	Pathogen-derived lncRNA	Regulate the motility of adult worms	[179]
	*SmLINC110998*	Pathogen-derived lncRNA	Regulate the motility of adult worms	[179]
	*Gm16685*	Host antisense lncRNA	Promote M1 macrophage polarization by regulating miR-205-5p	[183]
	*H19*	Host intergenic lncRNA	Influence S. japonica infection via *H19*/miR-130b-3p/*Cyp4a14* axis	[184]
*Cryptosporidium*	*U90926*	Host intergenic lncRNA (peptide coding)	Regulate cell autonomous antiparasitic defense in a pro-parasitic manner	[186]
	*Nostrill*	Host intergenic lncRNA	Promote antiparasitic defense through regulating NF-κB p65	[187]
	*NR_045064*	Host intergenic lncRNA	Promote host defense against Cryptosporidium by modulating NOS2/CSF2	[188]
	*XR_001779380*	Host intergenic lncRNA	Relevant to anti-Cryptosporidium defense in a IFN- dependent manner	[189]
	*NR_033736*	Host intergenic lncRNA	Contribute to host innate defense against *Cryptosporidium*	[190]
*Toxoplasma*	*Csf1-lnc*	Host sense overlapping lncRNA	Controlled by secretory kinase ROP16	[192]
	*Socs2-lnc*	Host sense overlapping lncRNA	Controlled by secretory kinase ROP16	[192]
	*lncRNA147410.3*	Host antisense lncRNA	Affect microglial proliferation, differentiation and apoptosis by targeting Hoxb3	[193]
	*lncRNA-11496*	Host sense overlapping lncRNA	Affect microglial proliferation, differentiation and apoptosis by targeting Mef2c	[194]
	*NONSHAT022487*	Host antisense lncRNA	Suppress the expression of the immune-related molecule UNC93B1	[195]
*Echinococcus*	*lncRNA028466*	Host intergenic lncRNA	Be involved in cytokine expression of Th1 and Th2	[200]

## 5. Conclusions

**Figure 1:**
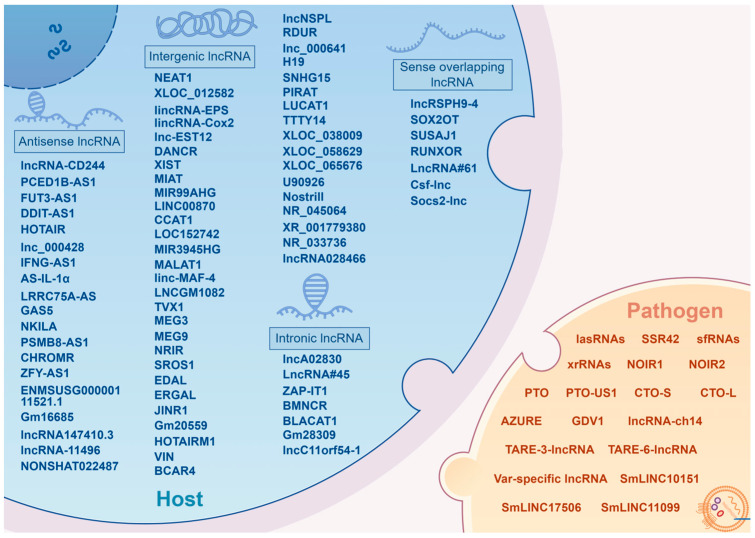
A brief summary of lncRNAs as a regulatory factor affecting zoonotic diseases (By Figdraw version 2.0, www.figdraw.com).

Microorganisms are ubiquitous. Zoonotic microorganisms, in particular, continue to cause communicable diseases with high incidence rates and result in considerable mortality. Mechanically, the spillover of these microorganisms typically involves breaching host barriers, inducing and evading innate immune responses, and surviving and proliferating within the host [6,201,202]. To our knowledge, these necessary processes to achieve spillover have not been systemically connected or elaborated for zoonotic pathogens. This review focuses on the roles of lncRNAs in the pathogenesis of zoonotic diseases. It also addresses the question of if lncRNAs are employed by pathogens to manipulate host immune status to their own advantage (Figure 1).

**Figure 2:**
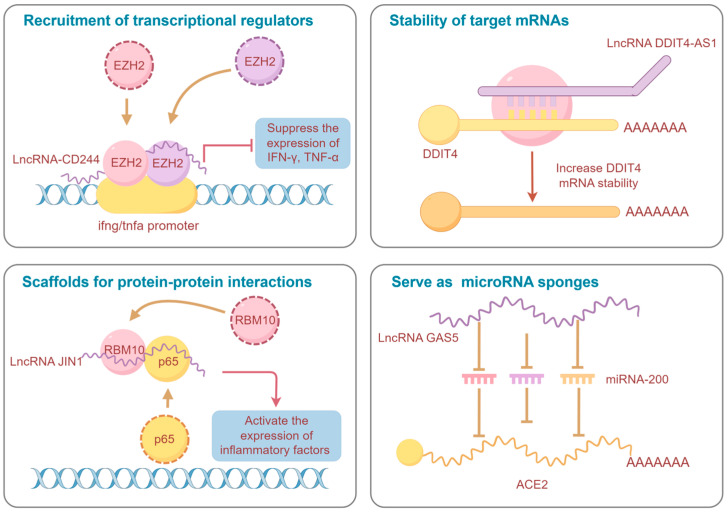
Schematic diagram of the regulatory mechanisms of representative lncRNAs in zoonoses (By Figdraw version 2.0, www.figdraw.com).

It is well known that lncRNAs are implicated in pathogen–host interaction by modulating host innate immune pathways. Several functional lncRNAs have been characterized in zoonotic infections, with the revelation of their involvement in pathogen invasion collectively contributing to the creation of a multidimensional regulatory network of lncRNAs in zoonoses (Figure 2). Among these, several broad-spectrum regulatory lncRNAs, such as *NEAT1*, *MALAT1*, *XIST*, *IFNG-AS1*, *lincRNA-EPS*, *DANCR*, *GAS5*, and *H19*, are identified as being implicated in a variety of zoonotic diseases [41,42,54,63,67,79,80,113,119,128,139,159,177]. Notably, these widely recognized functional lncRNAs exhibit a degree of conservation, holding potential as neoteric diagnostic markers and therapeutic targets. A prominent example is the *NEAT1*, which exhibits upregulation in most infections and assumes crucial anti-viral functions, thereby potentially serving as a therapeutic target for antisense and small molecule RNA inhibitor approaches. Conversely, the specific depletion of *NEAT1* has been associated with severe hemorrhagic fevers, suggesting its potential utility as a diagnostic biomarker [106].

Furthermore, this review also synthesizes the findings on pathogen-derived lncRNAs that display distinct characteristics in bacteria, viruses, and parasites. Specifically, lncRNAs are identified in zoonotic bacteria (e.g., *L. monocytogenes*, *P. aeruginosa*), viruses (e.g., EBV, *Flaviviridae* family viruses), and parasites (e.g., *Plasmodium*, *Schistosoma*) [84,89,121,152,170,179]. These pathogen-derived lncRNAs typically regulate both pathogenicity and the life cycle of the pathogens, leading to altered invasive behavior and potentially influencing pathogen spillover events. However, the current exploration and comprehension of lncRNAs within pathogens remains inadequate. Developing a more comprehensive lncRNAs map at the pathogen level is imperative to enhancing the identification of diagnostic and therapeutic targets, heralding promising future directions for lncRNA research of zoonotic diseases and even infectious diseases in general.

## Data Availability

Not applicable.
